# A Bayesian spatial model for neuroimaging data based on biologically informed basis functions

**DOI:** 10.1016/j.neuroimage.2017.08.009

**Published:** 2017-11-01

**Authors:** Ismael Huertas, Marianne Oldehinkel, Erik S.B. van Oort, David Garcia-Solis, Pablo Mir, Christian F. Beckmann, Andre F. Marquand

**Affiliations:** a Unidad de Trastornos del Movimiento, Servicio de Neurología y NeurofisiologíaClínica, Instituto de Biomedicina de Sevilla (IBiS), Hospital UniversitarioVirgen del Rocío/CSIC/Universidad de Sevilla, Seville, Spain; b Department of Cognitive Neuroscience, Radboud University Medical Centre, Nijmegen, The Netherlands; c Donders Centre for Cognitive Neuroimaging, Donders Institute for Brain, Cognition and Behaviour, Radboud University, Nijmegen, The Netherlands; d Servicio de Medicina Nuclear, UDIM, Hospital UniversitarioVirgen del Rocío, Seville, Spain; e Centro de Investigación Biomédica en Red sobre Enfermedades Neurodegenerativas (CIBERNED), Spain; f Oxford Centre for Functional Magnetic Resonance Imaging of the Brain (FMRIB), University of Oxford, United Kingdom; g Department of Neuroimaging, Centre for Neuroimaging Sciences, Institute of Psychiatry, King's College London, United Kingdom

**Keywords:** Multivariate GLM, Functional parcellations, Spatial statistics, Basis functions, Spatial statistics, Dopamine transporter SPECT, Parkinsonian disorders

## Abstract

The dominant approach to neuroimaging data analysis employs the voxel as the unit of computation. While convenient, voxels lack biological meaning and their size is arbitrarily determined by the resolution of the image. Here, we propose a multivariate spatial model in which neuroimaging data are characterised as a linearly weighted combination of multiscale basis functions which map onto underlying brain nuclei or networks or nuclei. In this model, the elementary building blocks are derived to reflect the functional anatomy of the brain during the resting state. This model is estimated using a Bayesian framework which accurately quantifies uncertainty and automatically finds the most accurate and parsimonious combination of basis functions describing the data. We demonstrate the utility of this framework by predicting quantitative SPECT images of striatal dopamine function and we compare a variety of basis sets including generic isotropic functions, anatomical representations of the striatum derived from structural MRI, and two different soft functional parcellations of the striatum derived from resting-state fMRI (rfMRI). We found that a combination of ∼50 multiscale functional basis functions accurately represented the striatal dopamine activity, and that functional basis functions derived from an advanced parcellation technique known as Instantaneous Connectivity Parcellation (ICP) provided the most parsimonious models of dopamine function. Importantly, functional basis functions derived from resting fMRI were more accurate than both structural and generic basis sets in representing dopamine function in the striatum for a fixed model order. We demonstrate the translational validity of our framework by constructing classification models for discriminating parkinsonian disorders and their subtypes. Here, we show that ICP approach is the only basis set that performs well across all comparisons and performs better overall than the classical voxel-based approach. This spatial model constitutes an elegant alternative to voxel-based approaches in neuroimaging studies; not only are their atoms biologically informed, they are also adaptive to high resolutions, represent high dimensions efficiently, and capture long-range spatial dependencies, which are important and challenging objectives for neuroimaging data.

## Highlights

• A multivariate spatial model using brain parcellations as basis functions is proposed.
• Brain regions can be modeled as a superposition of multiscale basis functions.
• These basis functions are biologically meaningful and capture spatial dependencies.
• Our framework allows to develop accurate and parsimonious clinical models.
• The model is computationally efficient, enhances power and adapts to high resolutions.

## 1 Introduction

Neuroimaging techniques have become invaluable tools for clinical research and practice in many brain disorders thanks to their ability to noninvasively investigate brain structure and function with relatively high spatial resolution. Data acquisition techniques such as MRI and PET allow the rich spatial structure that emerges from interactions between brain regions to be probed in high detail. However, the predominant analysis approaches that rely on the voxel as the unit of analysis do not take full advantage of this source of information. In the classical mass-univariate approach, which entails fitting independent temporal models at each sampled brain location (i.e. each voxel), spatial dependencies are effectively disregarded or dealt with suboptimally (e.g. by smoothing the data). This ignores an important source of information encoded by statistical dependencies between brain regions. The mass-univariate approach also generates a large number of statistical estimates that depend arbitrarily on the voxel size in the image. These spatially uninformed estimates need to be combined and inferred upon using complex post-hoc correction methods such as random field theory (Nichols, 2012, Worsley et al., 1996), the accuracy of which has been recently called into question (Eklund et al., 2016). Voxel-based features are also potentially suboptimal for multivariate approaches such as pattern recognition (Wolfers et al., 2015, Mwangi et al., 2014) essentially because voxels lack biological meaning. While pattern recognition approaches can make use of correlations between brain regions, the nature of neuroimaging data often leads to severely ill-posed problems (e.g. with hundreds of thousands of features and tens to hundreds of samples). Therefore, whole-brain voxel-based approaches are not optimal for discriminating conditions if the underlying signal is localized to particular regions or networks. For multivariate approaches as well as mass-univariate approaches it is therefore desirable to find parsimonious representations of brain structure or function that can more faithfully represent the underlying signal. Such models may predict clinically-relevant outcomes more accurately than voxel-based approaches and may be more interpretable in the sense that discriminating features may be cleanly related to underlying neuronal units of computation.

In light of these considerations, there have been some proposals to take spatial dependencies into account using multivariate approaches, and the field of spatial statistics offers attractive methods in this respect. Various discrete spatial models have been proposed for neuroimaging data (e.g., Penny et al., 2005, Woolrich et al., 2004) but these generally only provide local smoothing for the parameter estimates from mass-univariate analysis. They do not accommodate long-range dependencies that are intrinsic to neuroimaging data, nor overcome the arbitrary dependence on voxel size or the intricate structure-shape relationships of the brain. A more accurate and flexible approach is the spatial mixed model, in which an additional term, called a spatial random effect, is added to the model. Here, spatial dependencies are typically modeled using a continuous (usually Gaussian) spatial random field. The covariance matrix of this term describes the spatial correlation between allocations (e.g., voxels), and the inversion of this matrix is necessary to obtain suitable estimates under this model (Wikle and Royle, 2002). The immediate problem of applying this approach to neuroimaging data is the computational burden of this matrix inversion. Accordingly, this approach has principally been used in the context of restricted regions of interest (Bowman et al., 2008, Groves et al., 2009) although some studies have made use of data reduction techniques to approximate the underlying spatial process (Hyun et al., 2014, Zhu et al., 2014). An efficient alternative to model high-dimensional spatial processes is the use of low rank models, in which the covariance matrix is approximated by a reduced number of basis functions (Cressie and Johannesson, 2008). Most commonly, these basis functions are taken to be nonlinear functions, such as radial basis functions (RBFs), b-splines, or wavelets, that are placed all over the spatial domain. In spatial applications, multiple resolutions are typically used to capture both short and long ranges of spatial dependencies.

In this work, we introduce a spatial statistical modelling framework that uses data-driven basis functions to model neuroimaging data. These basis functions are derived from measures of brain function, and therefore more closely reflect the underlying biology relative to generic basis functions. While various spatial basis sets are possible, we propose to use a soft multiscale functional parcellation derived from resting-state fMRI (rfMRI). For this, we employ a parcellation strategy known as Instantaneous Connectivity Parcellation (ICP, van Oort et al., 2016). Our rationale is based on emerging evidence of temporally independent, spatially overlapping, subnetworks within anatomical regions and functional networks in the human brain (Smith et al., 2012). These subnetworks are believed to represent fine-scale units of computation used by the brain for processing. We use these subnetworks as basis function because of their correspondence with biology. There are various strategies that we could employ to extract these subnetworks (e.g., Yeo et al., 2011, Craddock et al., 2012, Shen et al., 2013, Gordon et al., 2016, Glasser et al., 2016), but the ICP approach is well suited to deriving such subnetworks as it combines three features: first, ICP sub-divides brain networks on the basis of fine-grained temporal similarities instead of temporally averaged correlations. Second, ICP does not impose a spatial contiguity constraint, meaning that brain regions that are not spatially adjacent can still participate in the same subnetwork. Finally, ICP follows a top-down strategy for parcellation, which generates sets of parcels at different levels of granularity which allows us to model multiple ranges of spatial dependencies in the image. We compare this approach to a variety of basis sets including: i) generic isotropic bisquare functions commonly used in spatial applications (Cressie and Johannesson, 2008); ii) structural parcellations of the striatum derived from two different atlases; and iii) functional parcellations of the striatum obtained from Independent Component Analysis (ICA).

For model fitting, we propose to use a Bayesian regression framework to automatically find a linearly weighted sum of basis functions that accurately fits an imaged brain region (or to the whole brain). The resulting basis function fit and the corresponding weights can be used in a second level of analysis to investigate the phenotype of the imaged subjects. To illustrate, we test our framework to predict quantitative SPECT data of the dopamine transporter (DAT) availability in the healthy striatum. DAT imaging allows assessing the integrity of presynaptic dopaminergic neurons of the nigrostriatal pathway and it is widely used in the clinical practice of movement disorders (Tatsch and Poepperl, 2013). We provide an example of how this method can be applied to a real clinical application. For this, we use the DAT data to automatically differentiate between different diagnosed sub-cohorts corresponding to different parkinsonian disorders. We hypothesized that spatial models that are informed by brain function would be superior to spatial models that are informed only by the structural anatomy and to generic models that do not incorporate knowledge of the underlying biology. Therefore, we compare functionally informed basis functions derived from resting state fMRI to anatomical basis functions derived from two widely used anatomical parcellations of the striatum and also to generic basis functions commonly used in spatial applications. The clinical application we have chosen provides an exacting test of this hypothesis for three reasons: (i) the spatial resolution of SPECT is low relative to alternative methods (e.g. fMRI) meaning that clinically relevant spatial dependencies are difficult to detect; (ii) anatomical subdivisions are well-defined for the striatum, which biases the analysis in favour of anatomical parcellations and (iii) the data modality used to create the basis set (BOLD fMRI, indirectly measuring oxygen consumption) measures different aspects of the underlying biology relative to the clinical biomarker (DAT SPECT, measuring dopamine function). Therefore the method must learn dependencies that generalize across different aspects of brain function.

Our approach is related to several lines of work in the neuroimaging literature. Gershman et al. (2011) developed a spatial modeling approach for neuroimaging data, referred to as topographic latent source analysis (TLSA). In TLSA, fMRI data are modeled as a superposition of image sources constructed from adaptive RBFs. Like our approach, TLSA abstracts away from the voxel as a unit of analysis, instead performing inferences over underlying neuroanatomical regions. However, in TLSA generic isotropic RBFs are used that do not map cleanly onto their biological sources (i.e. brain nuclei). The approach also requires running heavy optimization machinery in order to fit a given data set. Our approach is also related to dictionary learning approaches (e.g. Varoquaux et al., 2011) and to approaches that model neuroimaging data using multi-scale parcellations (e.g. Jennaton et al., 2012, Bellec, 2013). These approaches generally aim to segment a set of neuroimaging data into subject-specific or group level atlases. In contrast, our approach focuses on enabling statistical inference using various candidate basis sets. This is useful in many different contexts, including: (i) improving the accuracy of models that predict brain structure or function from clinical or demographic data (e.g. Marquand et al., 2016) (ii) abstracting away from the voxel as the unit of analysis which may lead to a lower multiple comparisons penalty in mass-univariate analysis, or to more accurate multivariate prediction of psychometric or clinical variables from neuroimaging data; (iii) adjudicating between different candidate basis sets or parcellations by providing a means to compare which most accurately explains the data at hand.

Our spatial model is generic, and can be adapted to investigate many different brain regions and research questions. Moreover, the proposed methodology provides four additional benefits: (i) biological interpretability of the computation units in the analyses (ii) a substantial reduction in the number of parameters for making inferences in neuroimaging studies, which consequently reduces correction penalties and enhances power; (iii) a faithful representation of the complex spatial structure of neuroimaging data in low dimensions and (iv) a quantification of the uncertainty in the predictions thanks to the Bayesian nature of the approach. In this work we demonstrate the validity of ICP basis set to make inferences in functional neuroimaging. Importantly, the multiscale nature of the ICP algorithm allows to efficiently capture the multiple ranges of spatial correlation in the brain. This enables to model spatially non-stationary correlation structures and long range dependencies in the data. These are both very challenging for classical spatial statistical models, yet are inherent properties of brain organization (Glasser et al., 2016).

## 2 Methods

### 2.1 Notational preliminaries

Throughout this section and what follows, we use bold lowercase characters to denote vectors (**a**), bold uppercase letters to denote matrices (**A**), plain letters to denote scalars (*A* or *a*), where we generally reserve lowercase letters for indexing and uppercase letters for fixed quantities.

### 2.2 Statistical model formulation

We use a flexible regression framework to model neuroimaging data in the spatial domain. To achieve this, we first reshape the preprocessed and masked three dimensional data volumes from each of *S* subjects into a vector $y_{s}$ of dimension *V*, where s=1,…,S. Our aim is to predict these data using a set of basis functions ${\left\{\phi_{m}\left(x\right)\right\}}_{m=1}^{M}$, that vary over the spatial domain, x, which for simplicity we take here to be coordinates in the Cartesian coordinate system. While these could be subject specific, here we employ a common set of basis functions across all subjects (described below). We consider that $y_{s}$ results from a linear combination *M* spatial basis functions plus a noise term:

$$y_{s}=\sum_{m=1}^{M}{\text{w}}_{m,s}\phi_{m}\left(x\right)+{\text{ε}}_{s}$$

where, $w_{s}={\left[w_{1,s},\ldots ,w_{M,s}\right]}^{T}$ is an *M*-dimensional vector of regression coefficients (weights) that are specific to each subject s and are adjusted to predict the class labels as accurately as possible. ${\text{ε}}_{s}$ represents additive Gaussian noise ${\text{ε}}_{s}∼N\left(0,\beta^{-1}\right)$ with β denoting the noise precision (i.e. inverse variance).^2^ In this paper, we cast this problem in the context of Bayesian hierarchical models, where prior distributions are placed over model parameters of interest. This provides several important benefits: most importantly, Bayesian models account for the uncertainty in the parameter estimates and provide implicit regularization of model parameters. They also provide a simple and elegant method to combine data from multiple subjects via a shared prior over the regression coefficients $\left(w_{s}\right)$ as outlined below.

In the first instance, we place a prior distribution over the regression coefficients only $\left(w_{s}\right)$. This yields a hierarchical generative model that can be succinctly summarized by the joint likelihood,^3^ which factorises in the following way:

(1) $$p\left(Y,\Phi ,W|\alpha ,\beta \right)=\prod_{s=1}^{S}p\left(y_{s}\text{|}\Phi ,\beta ,w_{s}\right)p\left(w_{s}\text{|}\alpha \right)$$

**Fig. 1:**
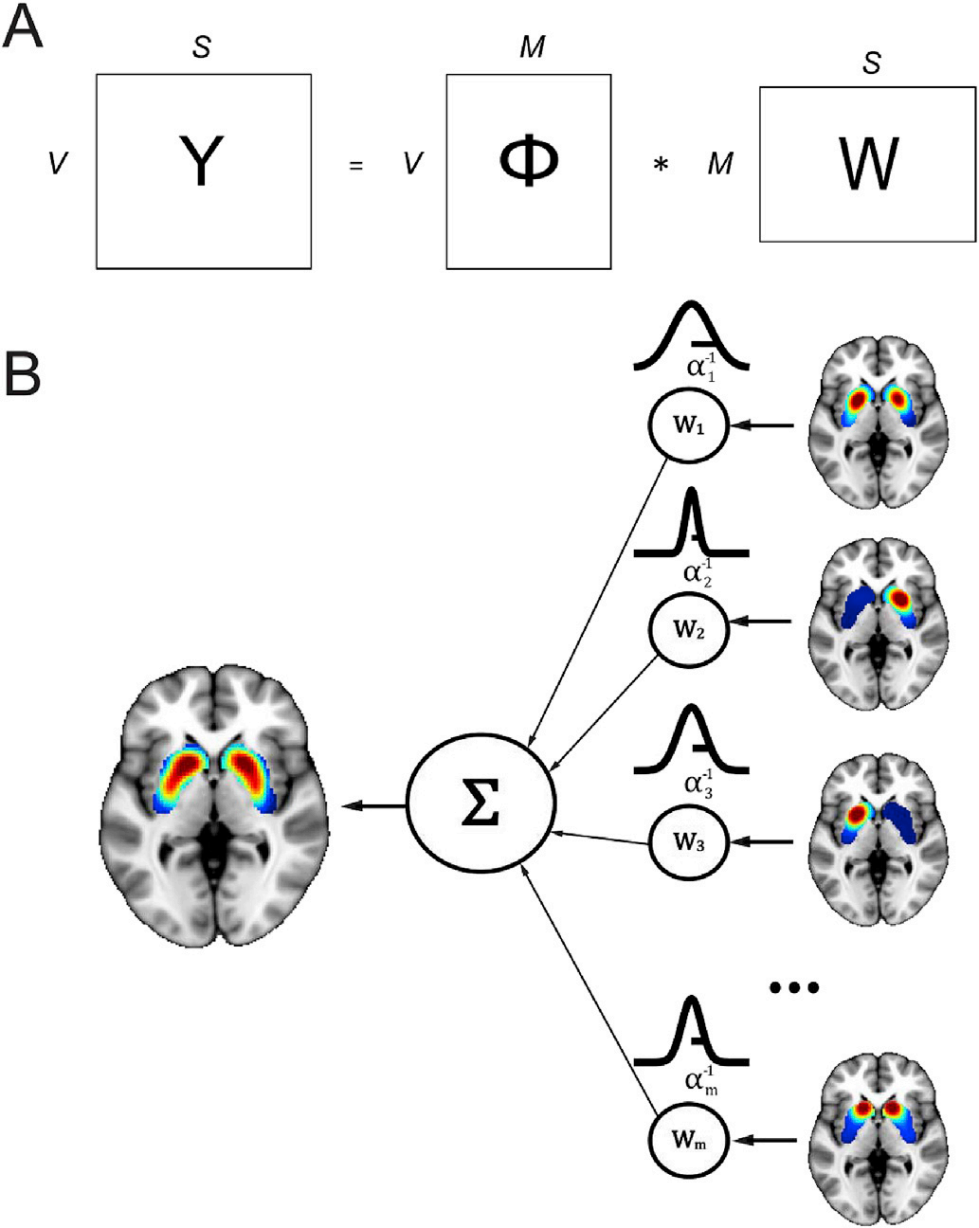
(A) The basic spatial model in matrix notation: the *S* neuroimaging vectors of dimension *V* (**Y**) result from a linear combination of *M* basis functions (**Φ**) and the corresponding weights (**W**). (B) Diagram of the model: DATSCAN images in the striatum are modeled as a superposition of *M* weighted striatal basis functions. A zero-mean Gaussian prior with precision $\alpha_{m}$ is placed over each weight, which determines the importance of each basis function for predicting the data.

Here, Φ is a V×M matrix that collects all the basis functions, $W=\left[w_{1},\ldots ,w_{N}\right]$ is an M×S matrix that collects the weight vectors for each subject and **Y** is a V×S matrix collecting the neuroimaging data for all subjects. We assume a Gaussian prior over the weights for each subject, such that $p\left(w_{s}\text{|}\alpha \right)=N\left(w_{s}|\mu ,\Lambda_{\alpha }^{-1}\right)$. Here, the precision matrix, $\Lambda_{\alpha }$ (inverse covariance matrix, i.e. $\Lambda_{\alpha }^{-1}=\Sigma_{\alpha }$), is shared across subjects and we make it explicit that it depends on a vector of hyperparameters ($\alpha ={\left[\alpha_{1},\ldots ,\alpha_{M}\right]}^{T}$). Without loss of generality, we also assume that the prior mean, μ, is zero. For the model in equation (1), the precision matrix is taken to be diagonal and is parameterized with an independent parameter for each basis function $\left(\alpha_{m}\right)$ along the leading diagonal. These parameters control the precision of each basis function, constituting an ‘automatic relevance determination’ prior (ARD; Mackay, 1995). Under this prior, the independent parameters for each basis function allow non-informative and redundant basis functions to be down-weighted and informative ones to be emphasized in a consistent manner across subjects. We could also take this one step further and apply priors over the precision parameters that further encourage them towards sparsity, which is the basis for the relevance vector machine (Tipping, 2001). However, we consider in our case that we do not have sufficient prior knowledge as to whether we should expect the model to be sparse. Therefore, we estimate the precision parameters from the model in equation (1) in an unconstrained manner, using an empirical Bayesian approach, described in the next section. The basic set up of this model is schematized in Fig. 1.

The model specified by equation (1) is appealing due to its simplicity, but it does not fully account for the uncertainty in the parameter estimates in that it places a prior distribution on the weight vector coefficients only. It also does not properly account for spatial correlations between basis functions. To address these problems, we employ a full Bayesian treatment of the problem, where we place prior distributions over all variables of interest and explicitly model correlations between basis functions. This gives rise to a hierarchical generative model in which the joint likelihood factorises in the following way:

(2) $$p\left(Y,\Phi ,W,\Lambda_{\alpha },\beta |\theta_{\beta },\theta_{\alpha }\right)=p\left(\beta |\theta_{\beta }\right)p\left(\Lambda_{\alpha }|\theta_{\alpha }\right)\prod_{s=1}^{S}p\left(y_{s}\text{|}X,\beta ,w_{s}\right)p\left(w_{s}\text{|}\Lambda_{\alpha }\right)$$

In this case, we have extended the generative model in equation (1) to accommodate correlations between the basis functions by allowing off-diagonal entries in $\Lambda_{\alpha }$ (and therefore also $\Sigma_{\alpha }$). We then place priors over the precision matrix of the ARD coefficients $\left(p\left(\Lambda_{\alpha }|\theta_{\alpha }\right)\right)$ and the noise precision $\left(p\left(\beta |\theta_{\beta }\right)\right)$ in addition to the weights, where $\theta_{\alpha }$ and $\theta_{\beta }$ denote the parameters of prior distributions for $\Lambda_{\alpha }$ and β. More specifically, we specify that the prior over the weights has the same Gaussian form as before: $p\left(w_{s}\text{|}\Lambda_{\alpha }\right)=N\left(w_{s}|0,\Lambda_{\alpha }^{-1}\right)$, the prior over the ARD precision matrix has a Wishart distribution $\;p\left(\Lambda_{\alpha }|\theta_{\alpha }\right)=Wish\left(\Lambda_{\alpha }|N,P\right)$ where N denotes the prior degrees of freedom and P denotes the prior precision.^4^ Finally, we specify that the prior over the regression coefficients has the form of a Gamma distribution $\;p\left(\beta \text{|}\theta_{\beta }\right)=Gam\left(\beta |a,b\right)$), where a and b are shape and rate parameters. This choice of priors greatly simplifies the inference in this model because it facilitates an efficient Gibbs sampling framework that capitalizes on the conjugacy of these distributions as described in Section 2.4.

### 2.3 Model estimation and inference: Empirical Bayes

For both of the models considered here (equations (1) and (2)), inference proceeds by estimating the posterior distribution over all parameters of interest. This is straightforward for the basic model specified in equation (1), because for fixed α and β the posterior distribution over W can be computed in closed form according to Bayes’ rule. For the model in equation (1), the posterior can be written as:

$$p\left(W|Y,\Phi ,\alpha ,\;\beta \right)=\frac{\text{ likelihood}\times \text{ prior}}{\text{marginal likelihood}}=\frac{\prod_{s}p\left(y_{s}\text{|}\Phi ,\;w_{s},\beta \right)\;p\left(w_{s}\text{|}\alpha \right)}{p\left(Y|\Phi ,\alpha ,\beta \right)}$$

It is straightforward to show (see e.g (Bishop, 2006)) that by combining a factorised Gaussian prior and Gaussian likelihood, the posterior is also a factorised Gaussian, such that $p\left(W\text{|}Y,\Phi ,\alpha ,\beta \right)=\prod_{s}p\left(w_{s}|y_{s},\Phi ,\alpha ,\beta \right)\text{.}$ The posterior weight vector for each subject ($w_{s}$) can then be written as:

(3a) $$p\left(w_{s}|y_{s},\Phi ,\alpha ,\beta \right)=N\left(w_{s}|{\bar{w}}_{s},A^{-1}\right)$$

(3b) $$A=\beta \Phi^{\text{T}}\Phi +\Lambda_{\alpha }$$

(3c) $${\bar{w}}_{s}=\beta A^{-1}\;\Phi^{\text{T}}y_{s}$$

Now, in order to calculate this posterior distribution, it is necessary to estimate optimal values for the hyperparameters α and β. For the model in equation (1), we achieve this using an empirical Bayes, or type-II maximum likelihood approach in which we work with point estimates of the hyperparameters (Bishop, 2006, Tipping, 2004). This is done by optimising the logarithm of the denominator of Bayes rule, namely the log marginal likelihood, with respect to the hyperparameters. The intuition behind this approach is that the marginal likelihood describes the probability of the data (Y) after integrating out the dependence on the parameters (W). As such, it embodies a tradeoff between model fit and model complexity and so by maximizing the marginal likelihood, one obtains an optimal balance between the two. In this case, the marginal likelihood can also be computed in closed form. This takes the following form, where we have taken advantage of the independence of subjects and have omitted the dependence on Φ for notational clarity:

(4) $$\log\;p\left(Y|\alpha ,\;\beta \right)=\log\int p\left(Y|W,\;\beta \right)p\left(W|\alpha \right)dW=\frac{SV}{2}\log\;\beta -\frac{SV}{2}\log\;2\;\pi -\frac{S}{2}\log\left|\Lambda_{\alpha }\right|-\frac{S}{2}\log\left|A\right|-\frac{\beta }{2}\sum_{s=1}^{S}{\left(y_{s}-\Phi {\bar{w}}_{s}\right)}^{T}\left(y_{s}-\Phi {\bar{w}}_{s}\right)-{\bar{w}}_{s}^{T}\Lambda_{\alpha }{\bar{w}}_{s}$$

To find α and β we employ a conjugate gradient optimization scheme as described in (Rasmussen and Williams, 2006). This requires the derivatives of the objective function given in equation (4), which can be found by applying standard identities for derivatives of expressions involving matrices and are given in the Appendix.

There are two key insights to note from the model specified by equations (1), (3a), (3b), (3c), (4). First, equation (1) embodies the assumption that subjects are independent realizations from the same distribution. This means that while the hyperparameters are shared across a group of subjects, the weights are estimated independently for each of S subjects. This provides a simple way to induce coupling between subjects via their shared reliance on a common set of hyperparameters. More generally, one could also employ multi-task learning (Bonilla et al., 2008, Caruana, 1997, Marquand et al., 2014) to couple the data from different subjects which does not require an independence assumption. However, this would be computationally costly, so we do not pursue it here. Second, equation (3a), (3b), (3c) shows that the posterior variance for the weights does not depend on the value of the response variables ($y_{s}$), only on the basis functions (Φ) and noise precision (β). Since we have chosen these to be fixed across subjects, this can lead to considerable computational improvements if this is accounted for in the implementation. In other words, it is not necessary to recompute the noise precision for each subject, only the posterior mean. For the remainder of this work, we refer to the approach where the model in equation (1) is fit using by optimising the objective function in equation (4) as ‘Empirical Bayes’.

### 2.4 Model estimation and inference: Full Bayes

For the model in equation (2), we adopt an alternative Markov chain Monte Carlo (MCMC) inference approach. This is highly desirable because it can accurately quantify the uncertainty over all variables in the model and allows a richer hierarchical model to be specified over the parameters. In more detail, we employ a blocked Gibbs sampling algorithm to estimate the full posterior distribution over quantities of interest, rather than point estimates. This is achieved by repeatedly sampling from the full conditional distribution of each block of variables conditioned on the current estimates of all the others. This breaks a complex, high-dimensional distribution into simpler, low-dimensional problems, which can be sampled by conventional methods. Moreover, we choose conjugate prior distributions for each block of parameters which means that the full conditional distribution for each block of parameters can be computed exactly and has a known distributional form, which makes them easy to sample. In more detail, for each of t=1,…,T iterations in the Markov chain, we draw samples from the full conditional distributions for W, β, and $\Lambda_{\alpha }$ based on the current estimates for the other parameters. This is achieved by repeatedly sampling from the full conditional distributions given below, where we use a superscript to denote the iteration number and again suppress the dependence on Φ:

(5a) $$p\left(W^{\left(t+1\right)}|\Lambda_{\alpha }^{\left(t\right)},\beta^{\left(t\right)},Y\right)=\prod_{s=1}^{S}N\left(w_{s}^{\left(t+1\right)}\text{|}{\bar{w}}_{s}^{\left(t+1\right)},{\left(A^{-1}\right)}^{\left(t+1\right)}\right)$$

(5b) $$p\left(\beta^{\left(t+1\right)}|\Lambda_{\alpha }^{\left(t\right)},W^{\left(t\right)},Y\right)=Gam\left(\beta^{\left(t+1\right)}\text{|}a+\frac{SV}{2},b+\frac{1}{2}\sum_{s}{\left(y_{s}-\Phi w_{s}^{\left(t\right)}\right)}^{T}\left(y_{s}-\Phi w_{s}^{\left(t\right)}\right)\right)$$

(5c) $$p\left(\Lambda_{\alpha }^{\left(t+1\right)}|W^{\left(t\right)},\beta^{\left(t\right)},Y\right)=Wish\left(\Lambda_{\alpha }^{\left(t+1\right)}\text{|}N+S,P+\sum_{s}w_{s}^{\left(t\right)}{\left(w_{s}^{\left(t\right)}\right)}^{T}\right)$$

For the remainder of this paper we will refer to the estimation of equation (2) using equation (5a), (5b), (5c) as ‘Full Bayes’. We chose vague top level priors for all models, such that a = b = 1, N = M + 2 and P = I. For each sampler, we check all posteriors samples for all model variables for convergence and efficiency by inspection of Markov chains and computation of diagnostic statistics (e.g. potential scale reduction factors (Gelman and Rubin, 1992)).

### 2.5 Computational complexity

While a detailed analysis of the computational complexity of the different inference methods is outside the scope of this work, it is nevertheless informative to make some brief remarks. For many applications, the computational cost of MCMC methods is high relative to alternative methods. In this case, however, the Full Bayesian approach based on MCMC compares very favourably to the alternative Empirical Bayesian approach. The overall computational cost of the Full Bayes approach is determined by the cost of computations per iteration, the number of iterations to achieve convergence and the number of samples collected for the posterior, which is in turn dependent on the autocorrelation in the samples in the Markov chain. In this application, equation (2) is linear in the parameters and conjugate priors we have employed mean that all variables can be readily sampled. As a result, the Gibbs sampling approach described in equation (5a), (5b), (5c) converges rapidly to the target distribution and is highly efficient in that successive samples in the Markov chains have low correlation for all sampled variables. In our experiments, a short burn in period (200 samples) was judged as sufficient to achieve convergence for all variables, and we used 1000 samples to estimate the relevant posterior distributions. For models with high numbers of basis functions the computational complexity is dominated by the need to compute the conditional posterior over W equation (5a), dominated by equation (3c), which has a cost of O(MVS) plus the cost of inverting the posterior covariance matrix ($O\left(M^{3}\right)$ in the worst case).

The computational cost of the Empirical Bayes approach is determined by the cost of making each iteration and the number of iterations to reach convergence. The Empirical Bayes approach is relatively efficient for small numbers of basis functions (e.g. the Oxford-Imanova and Harvard-Oxford basis sets described below), but the computational cost does not scale well to models with a large number of basis functions (e.g ICP, ICA and bisquare basis sets described below). In such cases, the computation is dominated by the cost of computing the derivatives of the marginal likelihood, which require computing multiple computationally expensive matrix products, many of which must be recomputed for every ARD parameter (see Appendix). The cost of computing the derivatives is over and above the cost for computing the posterior over W, which must still be computed at every iteration. In practice, for large problems (i.e. large M with V and S fixed) this means that the MCMC approach is usually an order of magnitude *faster* than the competing optimization approach.

### 2.6 Spatial basis functions

In this work we consider five approaches for constructing basis functions for the spatial model. These consisted of: two data-driven functional parcellations of the striatum based on (i) a recently developed instantaneous connectivity parcellation approach (van Oort et al., 2016) and (ii) a group-level independent component analysis (ICA); (iii) a set of generic basis functions widely used in spatial applications (Cressie and Johannesson, 2008) plus two anatomical parcellations of the striatum, derived from (iv) the probability maps derived from the structural MRI-based Harvard-Oxford (HO) atlas, and finally (iv) the DTI-based Oxford-Imanova (OI) atlas. Both anatomical atlases are available in FSL (http://fsl.fmrib.ox.ac.uk/fsl). These basis sets are described next and their most important characteristics are summarized in Table 1 below:

**Table 1:** Summary of the different basis sets evaluated in this work. The last column reports the mean (standard deviation) absolute value of the spatial correlation across all basis functions. For the functional basis sets (ICP and ICA), this value is after spatial smoothing (see Methods).

Name	Type	Data driven	Multi-scale	N basis functions	Correlation
ICP	Functional	Yes	Yes	464	0.34 (0.24)
ICA	Functional	Yes	No	464	0.21 (019)
Bisquare	Generic	No	Yes	681	0.02 (0.06)
HO	Structural	No	No	4	0.31 (0.19)
OI	Structural	No	No	7	0.36 (0.17)

#### 2.6.1 Instantaneous connectivity parcellation derived basis functions

We obtained a multiscale functional parcellation of the bilateral striatum by applying ICP to resting-state fMRI from 100 participants from the Human Connectome Project (HCP) (Van Essen et al., 2013), preprocessed using the HCP minimal processing pipelines (Glasser et al., 2013). Our rationale for using fMRI for estimating the basis functions using resting-state fMRI is that it provides a higher spatial resolution than SPECT, and therefore can potentially provide a richer characterization of the spatial structure of the functional architecture of the brain.

The ICP approach is described in detail elsewhere (Oldehinkel et al., 2016, Van Oort et al., 2016) but we provide a brief overview here. ICP is based on the assumption that voxels that form a subregion within a larger region exhibit similar, yet slightly different time courses compared to the other voxels in the larger region. The aim of ICP is therefore to divide the larger region into smaller, functionally homogenous sub-regions based on their temporal signature. The differences between these temporal signatures may be subtle, so in order to increase sensitivity for such differences, we analyse the dynamics of the ‘instantaneous’ modes of connectivity, reflecting the voxel-to-region differences in functional connectivity. In essence, we amplify the differences in (groups of) voxel time series by comparing them to a shared reference, here taken to be the grand mean average time course of the original region selected for parcellation.

Pearson correlation is the most widely used metric to quantify functional connectivity between brain regions or voxels. In such types of analysis, the measure of association is based on temporal averaging, which hides the rich dynamic information present in resting fMRI data. With ICP, we expand upon the basic Pearson correlation by considering the sequence of events across time. This proceeds by temporally ‘unfolding’ the time-averaged correlation between each voxel and the reference timeseries. For normalized timeseries (i.e. having zero mean and unit standard deviation) of length *T*, the Pearson correlation between time courses **a** and **b** can be written as the mean of the element-wise (Hadamard) product between them, i.e.:

$$\rho_{a,b}=\frac{1}{T}\sum_{t=1}^{T}a_{t}b_{t}$$

The essential intuition underlying the ICP method is that we analyse the time-resolved instantaneous connectivity between a regionally-specific reference time series and all voxels’ time series within the same region. In contrast to Pearson correlation, we do not perform temporal averaging over the quantity given above. This enables us to make use of the instantaneous temporal dynamics to sub-divide the original region into a set of subregions, based on the assumption that the temporal dynamics are also spatially structured. We derive a set of spatial modes describing this structure by feeding the temporally unfolded timecourse of each voxel with the reference timecourse into a group-level independent component analysis (ICA) as implemented in the FSL MELODIC software (Beckmann et al., 2004, Jenkinson et al., 2012). While we could also use this decomposition to derive a piece-wise constant parcellation (see van Oort et al., 2016), these are not well suited for use as spatial basis functions. Instead, we use a set of real-valued quantities describing the relative confidence by which each voxel can be assigned to each parcel (i.e. soft parcellation) which form the set of candidate basis functions (**Φ**) for our spatial model. These confidence measures are defined as the ratio between the probability of belonging to the alternative distribution, relative to the explicitly modelled null distribution (see Beckmann et al., 2004 for further details).

The ICP algorithm described above requires that the model order of the ICA decomposition be specified, although various approaches may be used to select the model order automatically (van Oort et al., 2016). In this work, we employ ICP do develop a multi-scale parcellation. Thus, for the striatum, we obtained subdivisions from model orders of $M_{d}=\left\{2,\ldots ,30\right\}=\left\{2,\ldots ,30\right\}$, generating a total of *M* = 464 basis functions $\left(\sum_{d=2}^{30}M_{d}\right)$.

#### 2.6.2 Independent component analysis derived basis functions

To act as a reference method, we compared the ICP method described above to a standard group-level ICA decomposition with a model order fixed to be equivalent to the ICP basis set above (*M* = 464). At such a high model order, ICA generates a large number of basis functions with generally very focal support (i.e., each having limited spatial extent). By comparing with the ICP basis set above, this allowed us to assess the importance of long-range interactions relative to local interactions in accurately modelling neuroimaging data. For this we employed the resting state data derived from the bilateral striatum from the same 100 subjects from the HCP dataset after the same preprocessing. We then estimated a group-level ICA from the concatenated data from all subjects and runs with the dimensionality fixed to *M* = 464. Note that we could also have employed ICP for this purpose, but we considered that at such a high model order, potential differences between the methods would be negligible.

#### 2.6.3 Generic local bisquare basis functions

As second reference method, we evaluated the ability of a generic basis set commonly used in classical spatial applications (Cressie and Johannesson, 2008). This involves tiling multi-resolutional basis functions all over the spatial domain to capture multiple ranges of spatial correlation (Cressie and Johannesson, 2008, Nychka et al., 2014). This reference method is therefore useful to assess the value of data-driven basis functions that aim to recapitulate the underlying biology with respect to basis functions that are simply multi-scale. Following Cressie and Johannesson (2008), we use local bisquare functions for this purpose. These take the form:

(6) $$\phi_{m_{d}}\left(x\right)=\{\begin{array}{ll} {\left[1-{\left(\frac{1}{r_{d}}\mathrm{||}x-c_{m_{d}}\mathrm{||}\right)}^{2}\right]}^{2}, & if\;\mathrm{||}x-c_{m_{d}}\mathrm{||}\leq r_{d}\\ 0 & otherwise \end{array}$$

Here, the $\phi_{m_{d}}\left(x\right)$ are the individual spatially-dependent basis functions, which are indexed by $m_{d}=1,\ldots ,M_{d}$ at the d-th detail level where again $M=\sum_{d}M_{d}$. The centres of each basis function are denoted by $c_{m_{d}}$ and $r_{d}$ denotes 1.5× the Euclidean distance between centre points at the d-th detail level. Intuitively, this basis function set can be considered as similar to a radial basis functions but with finite support across space. Here we choose three detail levels, having $\;r_{d}=\left\{6mm,\;12mm,\;18mm\right\}$, $M_{d}=\left\{589,\;72,\;20\right\}$ yielding a total of 681 basis functions. Note that the total model order is higher than the model order of the data-driven basis sets, but it was not possible to obtain an exact match because it is necessary to tile the entire space with basis functions.

#### 2.6.4 Anatomical basis functions

For the anatomical basis sets, we used the probability maps derived from: the 4 anatomical subdivisions (left and right putamen and caudate) from the MRI-based Harvard-Oxford (HO) atlas, and the 7 subdivisions from the connectivity DTI-based Oxford-Imanova (OI) atlas, supplied with the FSL software package v.5.0.9 (http://fsl.fmrib.ox.ac.uk/fsl).

**Fig. 2:**
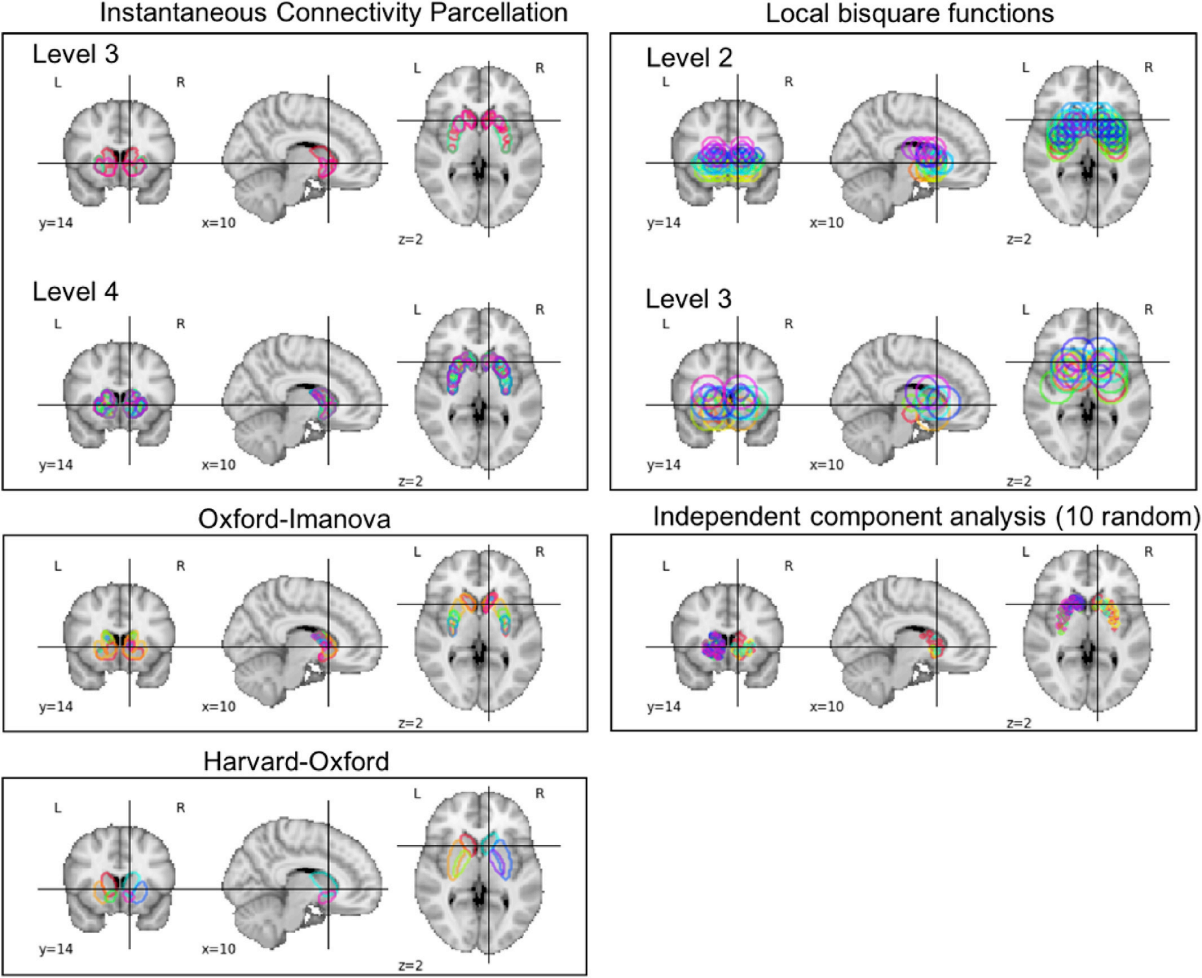
Basis functions used to model activity in the striatum. For the high-dimensional basis sets (independent component analysis, instantaneous connectivity parcellation and local bisquare functions), only examples are shown. Note also that the basis sets have not been masked to assist visualization.

We show examples of the different basis sets used to model activity in the striatum in Fig. 2. There are some characteristics that are worth commenting on: First, the soft nature of the parcellations fits with the idea that functional networks can be spatially overlapping (Smith et al., 2012). Thus, these parcellation schemes accommodate for the fact that one spatial unit may be involved in multiple, functionally relevant networks. With regard to the specific basis sets, the ICP and ICA basis sets are functional and data-driven and aim to derive the underlying units for the basis set on the basis of the underlying functional anatomy. They differ in that ICP provides a set of multi-resolution parcels, allowing brain units of varying sizes and with substantial spatial overlap to be combined to accurately model brain data. In contrast, the ICA basis set is derived from a single high-dimensional decomposition, so the parcels are all quite small and have lower spatial overlap. The local bisquare basis set does not use biology, but instead places basis functions across a regular grid and across multiple spatial scales. The anatomical basis sets are data-driven on the basis of structural anatomy, but are neither multi-scale nor functional. The intuition underlying these basis sets is that function to a certain extent recapitulates structure.

**Fig. 3:**
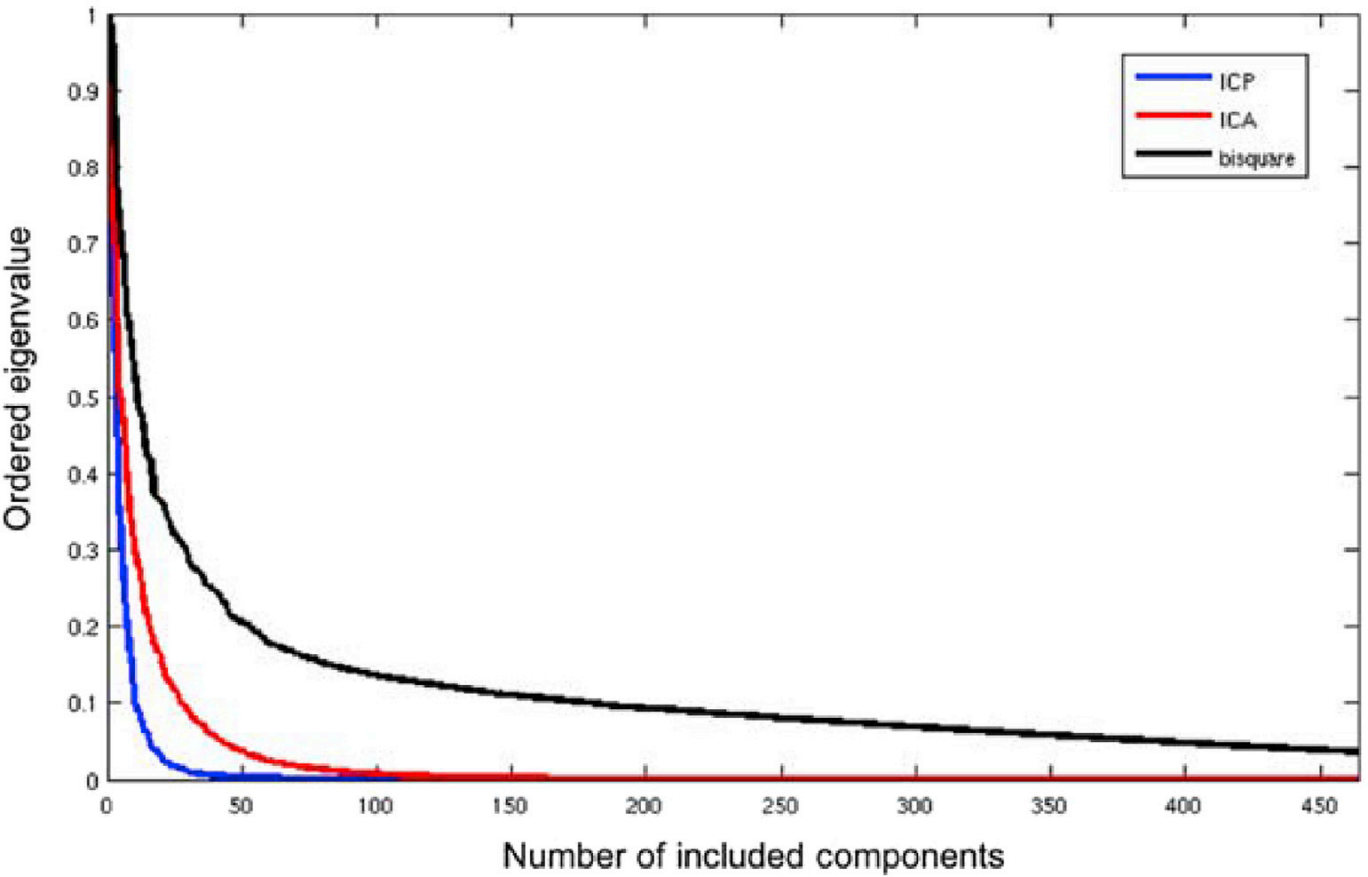
Eigenspectrum for the three high dimensional basis sets. Abbreviations: ICP = instantaneous connectivity parcellation; ICA = independent component analysis.

To quantify the spatial complexity of the different basis sets, we show normalized eigenspectra in Fig. 3 derived from performing an eigendecomposition of the ICP, ICA and bisquare basis sets separately. This shows that the ICP has a lower intrinsic dimension than either the ICA or bisquare basis sets (i.e. the eigenspectrum shows a greater proportion of energy in few basis functions. The bisquare basis set that does not use biology has the highest intrinsic dimensionality.

### 2.7 Correlation between features

It is apparent from Table 1 that there is high spatial correlation between the basis functions in the anatomical and data driven basis sets. This has consequences both for model estimation and interpretation of the coefficients. Collinearity between predictor variables (here, basis functions) makes model estimation more difficult – especially in high dimensions – because there are many possible linear combinations of features that can yield the same predictions. Collinearity also complicates the interpretation of the resulting regression coefficients (Kraha et al., 2012). There are two essential problems when covariates are highly collinear: (i) although unbiased, the regression coefficients have a high variance and can therefore be sensitive to slight variations in the data. This is again because there are many combinations of collinear covariates that can predict the data equally well. (ii) Care must be taken in the interpretation of high magnitude coefficients because a high magnitude coefficient can arise because a covariate is directly useful in predicting the data or because it acts as a ‘suppressor’ variable (Kraha et al., 2012); that is, that it helps to cancel out noise or mismatch in other covariates (Haufe et al., 2014). We perform two specific analyses to alleviate these concerns. First, we evaluate the reproducibility of the coefficients under different splits of the data, and second, we present structure coefficients that show the univariate correlation between the predictors and each covariate. These are a standard tool in linear regression models to assist interpretation of regression coefficients in the presence of collinearity (Kraha et al., 2012).

### 2.8 Model evaluation

We applied our spatial model to study dopamine function in the striatum as measured by DATSCAN, which is a reliable imaging test for the identification of striato-nigral degeneration. This scan is accurate and widely used in clinical practice for the diagnosis of Parkinson's disease (PD) and its differentiation from other movement disorders without presynaptic dopaminergic loss (e.g., essential tremor and drug-induced parkinsonism). However, the discrimination of PD from other parkinsonian disorders such as progressive supranuclear palsy (PSP) is much more challenging and current standard methods of assessments of image do not allow to make this differential diagnosis on the basis of DATSCAN images alone (Tatsch and Poepperl, 2013). It is even more challenging to discriminate putative subtypes of parkinsonian disorders from one another.

We provide two illustrative examples of this method in what follows. We first show a proof-of-concept example in which we use our method to obtain an accurate low-dimensional representation of the striatum using DATSCAN images of healthy controls (Section 2.8.1). Second, we provide a translational clinical example where we discriminate: (i) subjects with Parkinson's disease (PD) from healthy control subjects, (ii) PD subjects from progressive supranuclear palsy (PSP), which is a related parkinsonian disorder that is often misdiagnosed as PD in the early stages (Section 2.8.2) and iii) PSP subtypes from one another. The subtypes we considered were Richardson syndrome (RS) and pure akinesia with gate freezing (PAGF). These discrimination problems reflect important distinctions in clinical decision making.

#### 2.8.1 Low-dimensional representation of the healthy striatum

In this example we sought to develop a spatial model able to accurately fit the DATSCAN of healthy control (HC) subjects. We compared the model accuracy of this for all of the five candidate basis sets (ICP, ICA, bisquare, HO and OI).

##### 2.8.1.1 Subjects

We included a total of 100 subjects (52% males, 60 ± 7 years) reported as healthy by nuclear medicine specialists and who were scanned with [^123^I]FP-CIT SPECT at Hospital Virgen del Rocio, Sevilla, Spain. Details about the SPECT scanner and acquisition protocol can be found in a previous work (Huertas-Fernandez et al., 2014). SPECT images were spatially normalized into standard space using a custom template (http://www.nitrc.org/projects/spmtemplates). No smoothing was applied.

##### 2.8.1.2 Model set-up and evaluation

The bilateral striata of the scans were masked using a manually delineated region template of dimension *V* = 4622 (https://www.nitrc.org/projects/striatalvoimap). Data from the striata of the *N* = 100 healthy subjects were vectorised to form **Y**(*V*×*N*) and intensity standardized to have zero mean and unit standard deviation. Each of these is associated with an independent weight vector, collected in the matrix **W** (*M*×*N*) but were dependent on a common set of hyperparameters as described above. We formed **Φ** for each basis set (**Φ**_**ICP,**_ **Φ**_**ICA,**_ **Φ**_**bisquare**_, **Φ**_**HO,**_ **Φ**_**OI**_) and each of the functional basis sets were smoothed with an 8 mm full width at half maximum Gaussian kernel to emulate the point spread function of the SPECT scanner (Cot et al., 2004).

We evaluated the model performance by assessing the mean cross-validated explained variance. Since the primary goal of this work was spatial interpolation accuracy, we used a spatial subsampling method similar to approaches commonly used in other spatial applications (e.g. Hyun et al., 2014). For this we repeatedly retrained the model using 10 random subsamples of the data such that either 10, 20 or 50% randomly selected voxels were available for training and the remainder were used for testing.

Having established the generalizability of each of these regression models, we then vary the number of basis functions included in the model (such that *M*′ ≪ *M*). While there are various possible heuristics to select which basis functions to include in the model, a natural and effective approach is to select basis functions on the basis of their ARD coefficients, which is also the strategy employed by the relevance vector machine (Tipping, 2001). We first compare the explained variance for models containing only the top 50 basis functions by this metric (derived from the whole dataset) then examine the explained variance across a range of model orders. For this, we ordered basis functions by relevance based on their ARD coefficient and successively added informative basis functions in order to construct a complexity/accuracy tradeoff curve. Thus, for *M′* = 1, the model included only the most relevant bases; for *M′* = 20, the model included the 20 top relevant bases, etc.

#### 2.8.2 Discrimination of parkinsonian disorders

In this example, we used our framework to build spatial models with the different basis sets (ICP, ICA, bisquare, HO and OI) to construct features for disease classification purposes. We applied spatial models to discriminate three decision problems that represent the most important clinical problems for parkinsonian disorders, namely to discriminate: i) healthy controls from PD; ii) PD (in early stages, see below) from PSP and iii) PSP subtypes from one another (RS vs. PAGF). We also computed the classification performance of aclassical voxel-wise classifier (i.e., using all striatal voxels) in order to have a non-spatial approach as a reference.

##### 2.8.2.1 Subjects

We included next to the 100 HC subjects described in the previous section, 100 patients diagnosed with PD (63% males, 63 ± 12 years); 50 of them in early stage (disease duration 3 ± 2 years) and the other 50 in late stage (disease duration 13 ± 5 years); and 53 patients diagnosed with PSP (73 ± 7 years; disease duration 3 ± 2 years). Forty-three of the PSP patients presented with the classical Richardson Syndrome (PSP-RS), whereas the other 10 presented with a pure akinesia and gait-freezing (PSP-PAGF) phenotype. The diagnosis of PD was made using the UK Parkinson's Disease Society Brain Bank clinical diagnostic criteria and the PSP patients were diagnosed and labeled based on established clinical criteria (Williams et al., 2005). All patients were also scanned at the same site (Huertas-Fernandez et al., 2014).

##### 2.8.2.2 Model set-up and evaluation

The model set-up pipeline was the same as for the previous example to form **Φ** for the different basis sets. We also formed the output matrix **Y** for the disease groups (i.e., **Y**_**PD**_ and **Y**_**PSP**_) as we did for the healthy controls in the previous section. The weights from these models then form features for the comparisons between normal controls and PD, PD (early stage) and PSP, and between the PSP subtypes (RS vs. PAGF).

##### 2.8.2.3 Classification method

We employed a Bayesian probit regression classifier with ARD priors for all comparisons (Albert and Chib, 1993). This involves combining a standard probit (or cumulative Gaussian) likelihood, with a Gaussian prior over the regression coefficients. If we write the binary class labels for subject s as $t_{s}\in \left\{0,1\right\}$, we can write the joint likelihood of this model by:

(7) $$p\left(t,W|\Lambda_{z}\right)=N\left(z\text{|}0,\;\Lambda_{z}^{-1}\right)\prod_{s=1}^{S^{\prime }}H{\left(z^{T}w_{s}\right)}^{t_{s}}{(1-H\left(z^{T}w_{s}\right))}^{1-t_{s}}$$

Here, t is a vector of length $\;S^{\prime }$ that collects the classification target values for all subjects included in the classification problem (note that $S^{\prime }\neq S$); H(a) is the cumulative Gaussian density evaluated at a. This serves as a response function that maps the real-valued regression values to the unit interval; W are the estimated weights derived from the previous analyses and $w_{s}$ is a weight vector from a given subject. $\;N\left(z\text{|}0,\;\Lambda_{z}^{-1}\right)$ is a Gaussian prior over the classification regression coefficients (**z**) where the precision matrix, $\Lambda_{z}$, is again diagonal with an ARD parameter over each basis function. These control the variance of the weights of the latent regression function in much the same way as in the regression models considered in the previous section. However, unlike the linear regression models considered above, there is no closed form solution for the posterior or marginal likelihood owing to the nonlinearity in the classification likelihood function. Since this model is equivalent to a linear Gaussian process model we therefore make a Gaussian approximation to the posterior density using the expectation propagation algorithm, which has been shown to yield excellent performance (Nickisch and Rasmussen, 2008). We refer the reader elsewhere for details (Rasmussen and Williams, 2006). We evaluate the generalizability of all classification models using stratified ten-fold cross-validation where we measured classification performance via the area under the ROC curve (AUROC).

## 3 Results

### 3.1 Performance of different basis sets as a function of model order

**Fig. 4:**
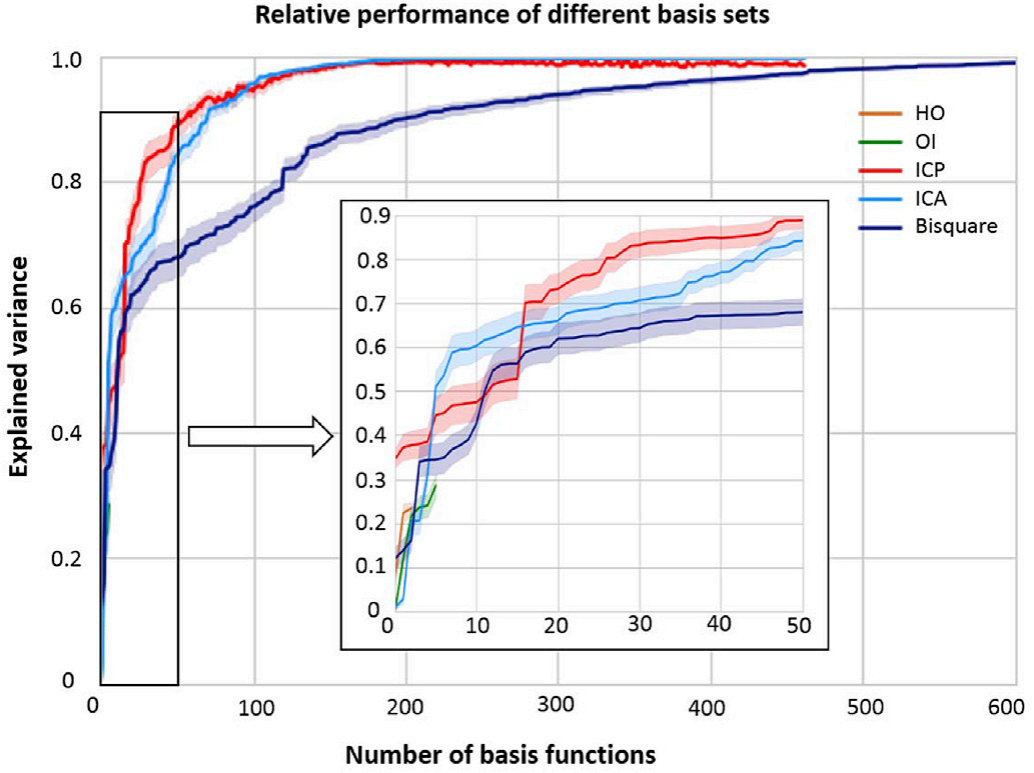
Explained variance (mean ± standard deviation across 100 subjects) as a function of the number of basis functions included in the model. Inset shows a zoom on the performance of all methods at low model orders.

We first chart the performance of the different basis sets as a function of the number of basis functions included in the model in terms of proportion of variance explained (Fig. 4). This plot was generated by sequentially adding basis functions to the model on the basis of their ARD coefficient estimated from the entire dataset. As discussed in the methods section, this provides a principled measure of the utility of each feature for predicting brain activity. For simplicity, these models were trained using the empirical Bayes approach although similar conclusions were reached using the full Bayesian approach. This shows that (i) all higher order basis sets (ICP, ICA and bisquare) predicted the DATSCAN data extremely accurately if a sufficient number of basis functions were included in the model, whereas (ii) the anatomical basis sets were substantially less accurate across nearly all model orders for which they were applied; Moreover, (iii) the data-driven basis sets (ICP and ICA) perform better than the generic basis set across most model orders, indicating that the data-driven basis sets give rise to more parsimonious models of brain function. This is important because for most applications it is crucial to derive a basis set that explains the data accurately *and* parsimoniously (i.e. using few basis functions). For example, it is reasonable to expect that more parsimonious models will lead to improved sensitivity in subsequent analyses. Finally, (iv) we note that there is a relatively small difference between the different data-driven parcellations although ICP outperforms ICA both at very low model orders (<5) and at moderate model orders (between 15 and 75 basis functions).

### 3.2 Performance of different inference algorithms for a fixed model order

**Fig. 5:**
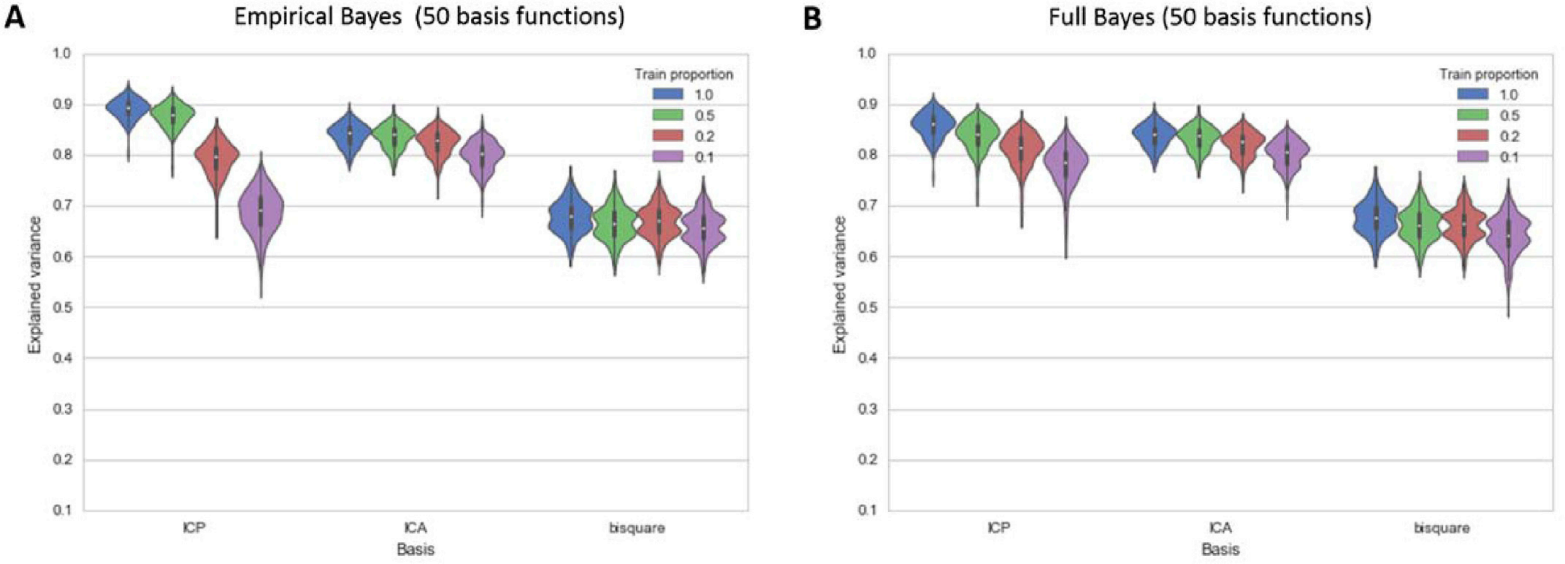
Total variance explained by the Empirical Bayes approach (A) and the Full Bayesian approach (B) for models using only the top 50 basis functions.

For the next analysis, we used a fixed model order of 50 basis functions which provides a reasonable trade-off between accuracy and computational complexity. Fig. 5 shows the performance of the high-order models on the basis of the 50 most informative basis functions according to their ARD coefficient. Similar to Fig. 4, this shows that at lower model orders the data-driven basis sets (ICP and ICA) dominate the generic bisquare basis set, where they explain approximately 15–20% more variance in the data.

Fig. 5 also shows that: (i) using the whole dataset (Train proportion = 1.0), the full Bayes and Empirical Bayes methods explain the DATSPECT data approximately equally accurately; (ii) as expected, the predictive performance of all methods drops as a smaller proportion of spatial data points are available to train the model (Train proportion < 1.0); (iii) for the ICP basis set, in which basis functions have high spatial correlation, the Empirical Bayesian approach overfits relative to the full Bayesian approach. To see this, observe that the out of sample explained variance decreases more rapidly under the empirical Bayes approach (Fig. 5A) relative to the full Bayes approach (Fig. 5B) as the proportion of training data decreases.

For completeness and to ensure that the choice of 50 basis functions was not biased toward the ICP basis set, we repeated the analysis using the entire set of basis functions (Supplementary Fig. 1). This lead to identical conclusions except that the degree of overfitting observed when combining the ICP basis set with the Empirical Bayesian approach was considerably more severe.

### 3.3 Interpretation of model coefficients

**Fig. 6:**
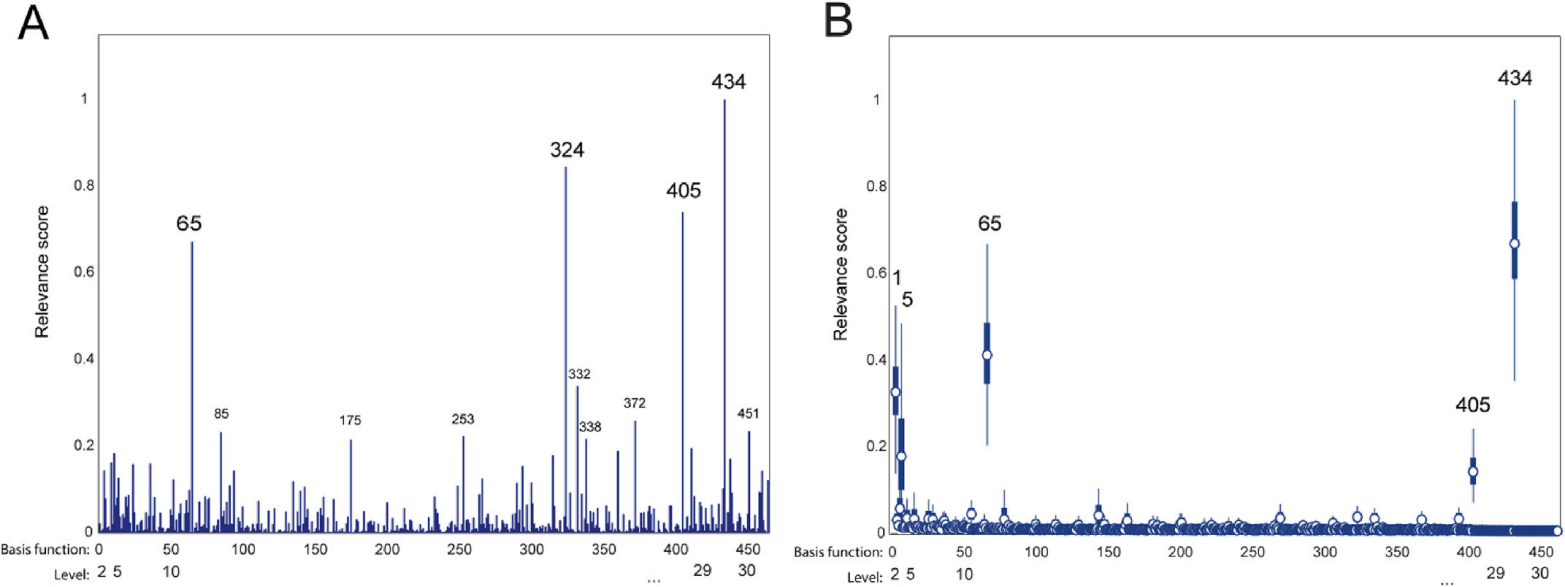
(A) Normalized relevance of the *M* weights using Empirical Bayes (B) Posterior variances of the weights using Full Bayes. Each weight correspond to a basis function obtained from instantaneous connectivity parcellation into *d* = {2,…,30} levels. These different levels of parcellation are denoted by bars along the x-axis.

An important benefit of this approach is to provide a low-dimensional representation of the data which can be readily interpreted with regard to underlying brain networks. To illustrate, we show the ARD coefficients from the ICP model in Fig. 6. In this case, the model produced a relatively sparse set of basis functions relevant for predicting striatal dopamine function. For visualization purposes, we show these by deriving a ‘relevance score’ from the Empirical Bayesian estimates (Fig. 6A), where we divide the absolute value of $\alpha_{m}$ for each basis function with respect to the maximum ($\alpha_{m}^{MAX}$). These largely correspond with the posterior variance derived from a full Bayesian model having a diagonal covariance matrix (Fig. 6B). This shows that there were relatively few basis function with high relevance (e.g., *m* = 1, 5, 65, and 434). Importantly, the top ranked basis functions had also high structure coefficients (Supplementary Fig. 2), and were highly consistent across cross-validation splits (r > 0.9) which confirms the relevance of these variables for the model and provides strong evidence against the possibility of these high coefficients being driven by suppressor effects.

**Fig. 7:**
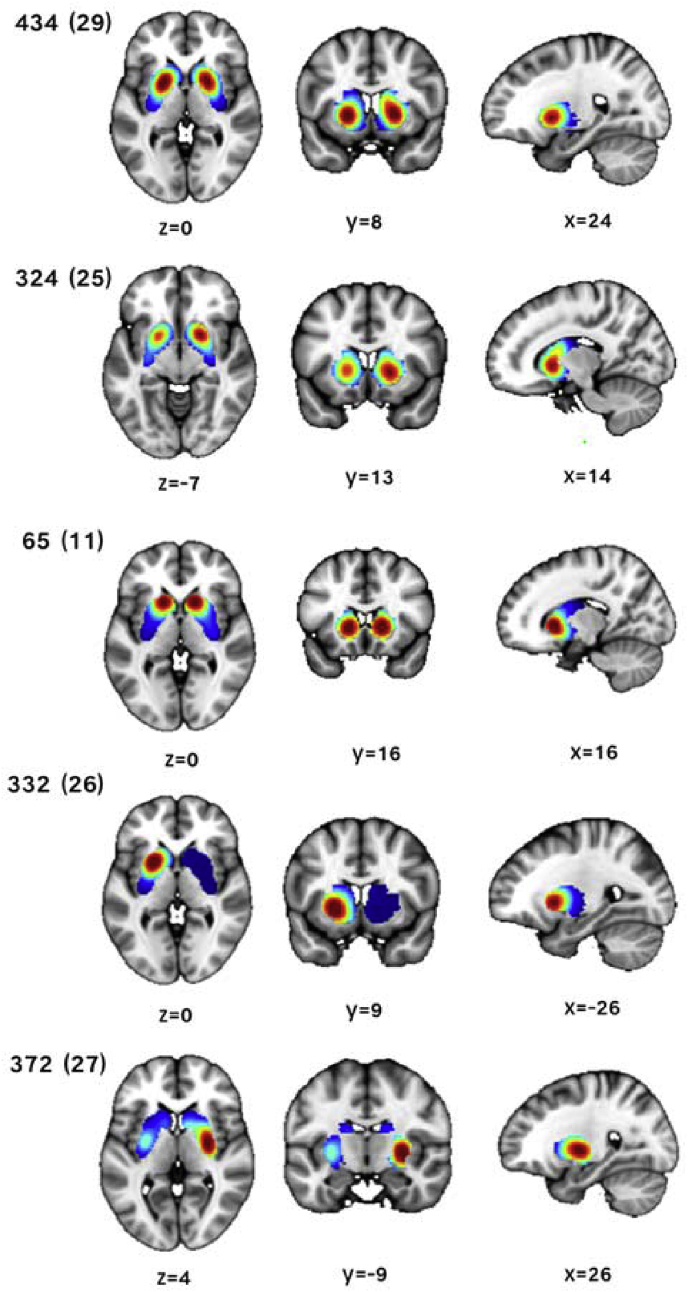
A selection of the top ranked basis function with coordinates given in MNI space. Notation: basis function number (level).

Fig. 7 illustrates that the top-ranked basis functions largely mapped different regions of the striatum and with different spatial length-scales (i.e. smoothness). For example, the basis functions 65 and 434 were spatially localised covering major regions of the caudate and putamen, respectively. Hence, the combination of these basis functions capture different spatial features and varying ranges of spatial correlation, respectively. Finally, note that there were relevant basis functions across multiple scales of parcellation and that not all of the top ranked basis functions were bilaterally expressed.

### 3.4 Discrimination of parkinsonian disorders

Finally, we compare the predictive capability of the different basis sets for diagnostic classification (PD vs HC), for differential diagnosis (PD vs PSP) and for separating subtypes of PSP (RS vs PAGF).

The AUROC for these classifiers using is shown in Table 2. These results show that – although not necessarily optimal for all comparisons – the ICP basis set produced good performance in all cases. In more detail, the discrimination between PD and healthy controls is an easy classification problem on the basis of DATSCAN images; all methods performed approximately equally well and at ceiling levels. The discrimination between PD and PSP is known to be very challenging using DATSCAN and this was reflected in our results. We obtained a moderate classification performance across basis sets where the voxel-based approach produced the highest accuracy, the ICA basis set performed relatively poorly and the other approaches were intermediate. For the classifiers trained to differentiate PSP subtypes (RS vs PAGF), the voxel-, OI- and ICA based approaches performed poorly while the ICP basis set performed well, with the other approaches were intermediate. This suggests that the benefit of spatial methods is dependent on the classification problem and the nature of the underlying pattern. Some classification tasks can be solved by non-spatial methods whereas other classification tasks benefit from methods that can capture subtle spatial differences. For example, voxel-based classifiers can work well if the signal is distributed across the whole region of interest (e.g. PD vs HC) whereas more subtle distinctions may be better approached by a more focused approach where one or more basis functions capture the salient differences (e.g. RS vs PAGF).

**Table 2:** Area under curve of classifiers trained to discriminate parkinsonian disorders.

Basis set	PD vs HC	PD vs PSP	RS vs PAGF	Mean
ICP	0.99	0.78	0.88	0.88
ICA	0.93	0.65	0.56	0.71
Bisquare	0.99	0.76	0.75	0.83
Harvard-Oxford	0.99	0.79	0.76	0.83
Oxford-Imanova	0.99	0.82	0.57	0.79
Raw voxels	1.00	0.85	0.42	0.76

**Fig. 8:**
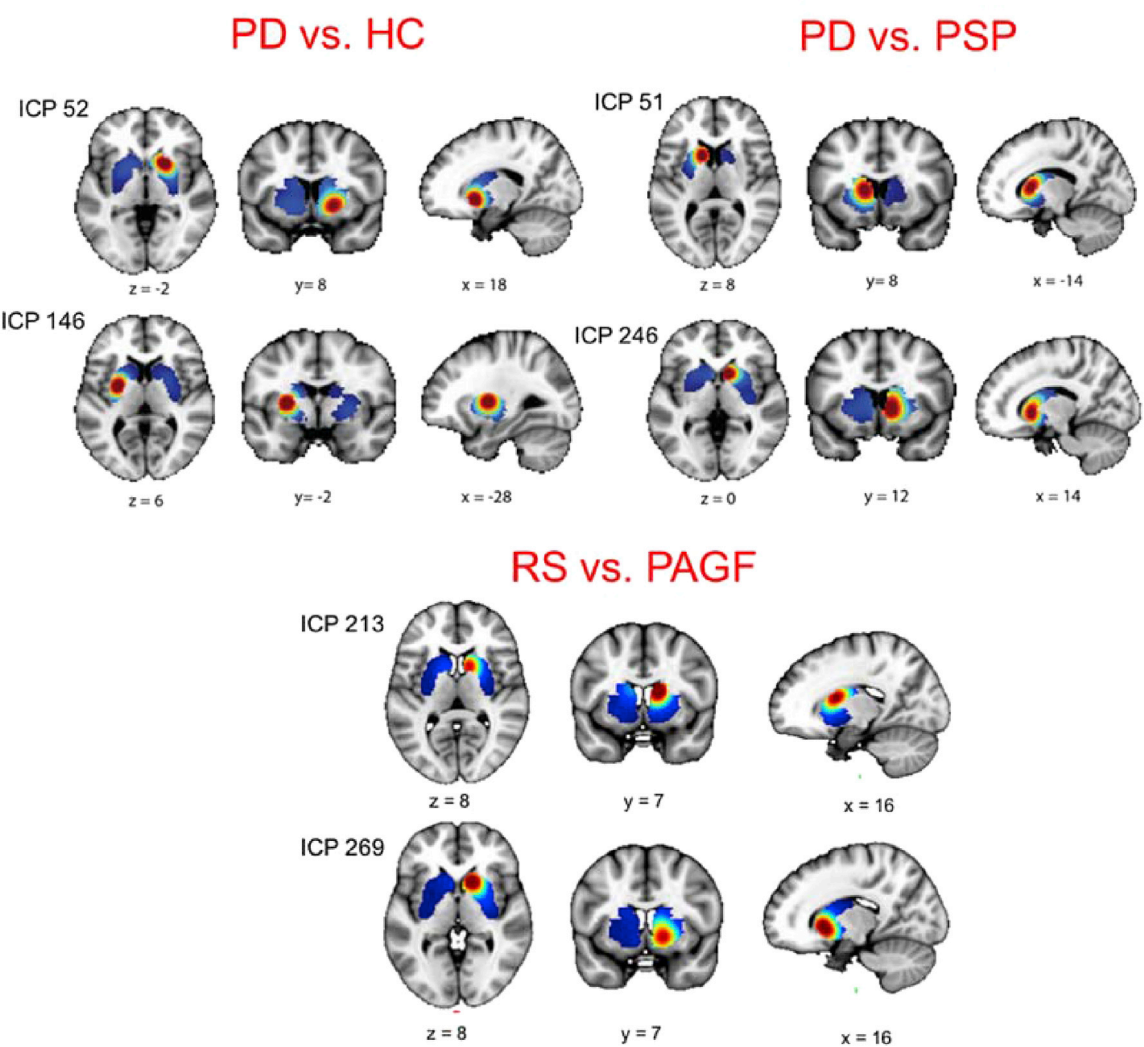
Representation of the top two ICP discriminative basis functions for each of the classifications considered in the text. Abbreviations: PD = Parkinson's disease; HC = healthy controls; PSP = progressive supranuclear palsy; RS = Richardson's syndrome; PAGF = progressive akinesia with gate freezing.

As noted, one of the benefits of using brain parcellations to build basis sets is the interpretability of the discriminative features. We illustrate in Fig. 8 two of the top-ranked discriminative ICP basis functions for each comparison. Overall, these are congruent with the known pathophysiology of these disorders which is important in the development of disease biomarkers in machine learning approaches. For example, the basis functions for distinguishing PD from NC were centered on the putamen and the ventral striatum whereas for PSP (vs. PD) were instead centered on the caudate.

## 4 Discussion

In this work, we presented a new spatial modeling approach for the analysis of neuroimaging data that entails characterizing spatially distributed effects as a linear superposition of multiscale functional basis functions. This framework provides an elegant alternative to classical voxel-based approaches and provides several advantages including: (i) a gain in interpretability since the units of analysis have a stronger biological basis relative to voxels or generic basis functions (van Oort et al., 2016); (ii) incorporation of multi-resolutional spatial information in the image, thus capturing not only local dependencies but also long range interactions; (iii) the ability to integrate information from multiple imaging modalities to derive more accurate or parsimonious models and (iv) a method to automatically identify meaningful subregions/subnetworks. This leads to several other practical advantages: for mass-univariate analysis this provides a great reduction of the number of statistical tests, leading to enhanced statistical power. For multivariate pattern recognition analyses, this may provide more accurate prediction of clinical outcomes because the derived basis functions provide a better match to the underlying neural computation units than voxel-based approaches or generic basis functions. Finally, our approach provides a method by which alternative parcellation approaches can be compared quantitatively. These properties enabled us to demonstrate that the ICP approach we employ to create basis functions provided more accurate models of brain function for a given model order than anatomical parcellation schemes predominantly used in the field and produced highly competitive performance in a clinical discrimination task relative to a range of competing approaches.

An important benefit of our approach is that it provides a full spatial statistical model for the observed imaging data. This enabled us to quantitatively compare different basis sets in terms of the accuracy with which they can predict the observed imaging data. For this, we found that the functional data-driven basis sets (ICP and ICA) performed better than structural basis functions, and ICP performed considerably better than structural basis functions at equivalent model order. Bisquare functions performed well with a large number of basis functions, but at an equivalent model order nearly always performed worse than the functional basis sets. Moreover, ICP performed slightly better than ICA across many model orders (Fig. 4). Taken together, these results allow us to draw the following conclusions: (i) functional basis functions explain function better than either structural or generic basis functions, even if the generic basis functions are capable of modeling multi-scale interactions; (ii) whilst long-range interactions are probably important (i.e. ICA performed worse than ICP across most model orders), this difference is probably less important than employing a basis set that is rooted in brain function. Finally, (iii) the fact that oxygen consumption (BOLD fMRI) helps to accurately explain dopamine function (DATSCAN) indicates that by taking advantage of networks that putatively reflect the true underlying biology of human brain function, information can be usefully combined across different imaging modalities. Nevertheless it is possible that there are aspects of dopamine function that we are not able to capture using resting fMRI (e.g. that are related to dopamine receptor distribution but not function as measured by resting fMRI), which may for example, put an upper bound on the accuracy obtainable in the classification experiments.

Whilst our approach is flexible and provides benefits both for univariate analysis and multivariate analysis techniques, one important application is for generating biomarkers for clinical discrimination problems. We illustrated this by using the coefficients of these spatial models as biomarkers for diagnostic classification, differential diagnosis and for discriminating subtypes of parkinsonian disorders. For diagnostic classification all approaches perform comparably to one another and at an accuracy that is highly competitive with existing benchmarks in the field (Bowman et al., 2016). Differential diagnosis and discriminating subtypes are both very challenging problems and it is therefore salient that our approach also performed well in those cases. Whilst not the most accurate in every condition, the ICP basis functions were the approach that performed best across all discrimination problems. Notably, the classical voxel based approach performed well in the diagnostic task where it is reasonable to expect that discriminating information is diffusely distributed across the whole striatum but performed very poorly for discriminating subtypes of parkinsonian disorders, where the discriminating information is probably restricted to select areas of the striatum. This suggests that for the more subtle distinctions it is more important to consider functional anatomy in developing biomarkers and reinforces the value of using functionally defined basis functions that map with biology for discrimination problems. Our results also provide evidence for a biological basis for the distinction between subtypes of PCP (i.e. RS vs PAGF), which often difficult on a clinical basis alone. This complements evidence from clinicopathological studies that have shown that RS and PAGF have different pathological burden and distribution in the brain (Williams and Lees, 2009).

We evaluated two different approaches for statistical inference in these models, and showed that in most cases a Full Bayesian approach is to be preferred; first, it produces similar performance in cases with small numbers of basis functions or when the basis functions are uncorrelated (e.g. the bisquare basis functions). In cases with high numbers of basis functions and strong correlations between basis functions, the Full Bayesian approach is more robust to overfitting than the competing Empirical Bayes approach. Second, and unlike many problems where MCMC methods are applied, it can be computed at a modest computational expense that is in many problem settings lower than what is required for the competing approaches. Finally, the full Bayes approach quantifies the uncertainty across all model parameters and propagates that uncertainty through to the predictions. This is important for application where predictive uncertainty is important. For example, in our previous work, predictive uncertainty is used to quantify variation across cohorts of participants (Marquand et al., 2016).

In this work we employ soft parcellations to construct a neural basis set, which provides several advantages over the common approach of hard partitioning the brain using clustering techniques. For example, soft parcellations mitigate the risk of mixing signals from different brain regions if the definition of the spatial parcels is inaccurate. They also allow one spatial unit to be involved in different networks (see e.g. Fig. 1) and for a more gradual transition in underlying organization. We combine this with a principled method to select the most informative basis given the data and the experimental question, and further show in that these subdivisions can not only more accurately represent brain activity relative to other parcellation methods but also have a clear correspondence with pathophysiological processes. For example, in line with the documented striatal uptake loss pattern in DATSCAN (Tatsch and Poepperl, 2013), we have seen functional parcellations located in the putamen that are discriminative for PD (vs. normal controls), and in the caudate for PSP (vs. PD). The weights associated with these basis functions can be used to investigate the association with phenotypic variables and may constitute a new potential avenue for the development of imaging biomarkers.

We elected to use a multivariate discrimination task to illustrate the value of our method but our approach is also beneficial for mass univariate analysis (which will be the topic of a follow-up report). For mass-univariate analysis, the reduction in the number of parameters is substantial with respect to voxel-based univariate approaches. For example, we were able to accurately model (R^2^ > 90%) the striatum of healthy controls with only *M* = 50 basis functions (*M* ≪ *V,* where *V* = 4622). An advantage of this reduction is a substantially lower multiple comparisons penalty and therefore a gain in statistical power. The number of basis functions and therefore the multiple comparison correction for univariate analyses will depend on the number of subdivisions conducted with ICP. For our example applications we subdivided the striatum into up to 30 parts, but this value can be different based on prior hypothesis or knowledge about the level of granularity of certain region or network. Van Oort et al. (2016) propose to use split-half reproducibility to learn about the optimal granularity of the parcellation. In any case, the number of subdivisions will always be much lower than the number of voxels so the gain in statistical power will always be substantial. This enables the detection of effects with smaller sample sizes. Also, in contrast to voxel-based approaches, the number of parameters and consequently, the multiple comparison penalty does not increase with spatial resolution. Indeed, brain images at higher spatial resolutions may yield spatially richer basis functions which may lead to more accurate predictions. In this application, the ICP basis set we chose was optimized for prediction, but for mass-univariate analysis it is probably advantageous to choose a basis set with lower correlation between basis functions. For this, a hierarchical approach is probably preferred, which is easily performed using the ICP method (van Oort et al., 2016).

In addition to being highly accurate, our method is computationally efficient and highly scalable relative to other spatial statistical approaches for neuroimaging data (Bowman et al., 2008, Hyun et al., 2014, Zhu et al., 2014, Gershman et al., 2011). For example, in TLSA (Gershman et al., 2011), activations are modeled using radial basis functions, each of which requires both location and spatial bandwidth parameters to be set resulting in many hyperparameters that have to be optimised given the data. In contrast, our set of basis functions have empirically-defined amplitudes and lengthscales and the optimisation step refers only to the hyperparameters of the weights and not to the configuration of the functions per se. Moreover, we expressly designed our approach to be able to scale to high-resolution whole-brain prediction. This is possible because the computational complexity is effectively governed by the number of basis functions, not the number of voxels. In contrast, the computational scaling of most of the other spatial modelling approaches that we are aware of is dependent on the number of voxels. Therefore, such methods are generally limited to regions of interest (e.g. Bowman et al., 2008, Groves et al., 2009, Hyun et al., 2014). Another important property of our method is the improved modeling of the spatial information contained in the image. The spatial correlation between locations, especially between distant voxels, is not properly modeled by voxel-based approaches. In this sense, the multiscale nature of ICP allows to capture both local spatial dependencies and long-range interactions, which can yield improved sensitivity relative to voxel-wise approaches (Bowman et al., 2008). This could be noted in PSP subtype classification example where the classifier using the raw voxels gave very poor performance, which may indicate that particular classification task required from richer spatial information to detect subtle differences. However, we have seen that ICP may not always be the best basis set for all the applications (e.g., it was not optimal for the PD vs. PSP comparison) and indeed we recommend to evaluate our spatial model with other types of basis set for further applications. For example, fine-grained parcels obtained from ultra-high resolution MRI can be used to develop spatial models for structural MRI (Iglesias et al., 2015, Keuken et al., 2014). Multi-modal parcellation methods (e.g. Glasser et al., 2016) are also good candidates for the basis set, although such parcellations are often not multi-resolutional, which is disadvantageous for modeling spatial dependencies across multiple scales. Another potentially promising approach is may make use of hierarchically defined whole-brain atlases (e.g. van Oort et al., 2014).

Finally, our approach is generic and is able to accommodate the most common types of designs and questions in neuroimaging studies. We provide a framework that can be easily applied to modeling groups of related scans so that studies involving case-control, multiple groups or task fMRI experiments can be easily accommodated. The weights (**W**) obtained can be used in further analyses to compare between groups or investigate quantitative measures with parametric statistics or machine learning techniques. This provides additional benefits to those noted above, including the ability to use information encoded by spatial correlation. We demonstrated the value and the flexibility of our approach by using it to construct classifiers with different basis sets that were able to accurately distinguish PD patients from controls. This degree of accuracy is comparable to what is obtained using current procedures in the diagnostic workflow of PD and other neurodegenerative parkinsonisms (e.g. putamen quantification; Tatsch and Poepperl, 2013) and automated diagnosis (Bowman et al., 2016), so this example is only intended to validate our method using a well-established clinical application. Furthermore, our approach can also be used to provide new insights into disease mechanisms. For example, it would be interesting to use our model to investigate the correlation between the degeneration of fine striatal subnetworks with specific symptoms in parkinsonism, such as rigidity, gait disorder or dyskinesias.

In summary, in this paper we presented a methodological framework for spatial modeling in neuroimaging with multiple advantages relative to existing approaches. In future work we would like to investigate other neuroimaging modalities and other brain regions. The framework we present is very generic and can be used to explore traits or symptoms in any brain disorder from a new perspective and has high potential to lead to methods that can be translated to real clinical practice.

## Conflicts of interest

CFB is director and shareholder in SBGNeuroLtd.

## Appendix

The derivatives of the log marginal likelihood with respect to the hyperparameters are given below

$$\frac{\partial }{\partial \beta }\log\;p\left(Y\text{|}\alpha ,\;\beta \right)=\frac{SV}{2\beta }-\frac{S}{2}\text{tr}\left[A^{-1}\Phi^{T}\Phi \right]\text{+}\sum_{s=1}^{S}y_{s}^{T}\Phi {\bar{w}}_{s}+\beta y_{s}^{T}\Phi b_{s}-\frac{1}{2}{\bar{w}}_{s}^{T}\Phi^{T}\Phi {\bar{w}}_{s}-\beta b_{s}^{T}\Phi^{T}\Phi {\bar{w}}_{s}\text{-}\frac{1}{2}y_{s}^{T}y_{s}-b_{s}^{T}\Lambda_{\alpha }{\bar{w}}_{s}$$

$$\frac{\partial }{\partial \alpha_{m}}\log\;p\left(Y\text{|}\alpha ,\;\beta \right)=-\frac{S}{2}\text{tr}\left[\Lambda_{\alpha }\frac{\partial \Lambda_{\alpha }^{-1}}{\partial \alpha_{m}}\right]-\frac{S}{2}\text{tr}\left[A^{-1}\frac{\partial \Lambda_{\alpha }}{\partial \alpha_{m}}\right]+\sum_{s=1}^{S}\beta c_{s}^{T}\Phi^{T}y_{s}-\beta c_{s}^{T}\Phi^{T}\Phi {\bar{w}}_{s}-c_{s}^{T}\Lambda_{\alpha }{\bar{w}}_{s}-\frac{1}{2}{\bar{w}}_{s}^{T}\frac{\partial \Lambda_{\alpha }}{\partial \alpha_{m}}{\bar{w}}_{s}$$

where:

$$b_{s}=\left(I-\beta A^{-1}\Phi^{T}\Phi \right)A^{-1}\Phi^{T}y_{s}$$

$$c_{s}=\beta \frac{\partial A^{-1}}{\partial \alpha_{m}}\Phi^{T}y_{s}$$

## Footnotes

2 Throughout this paper for convenience we work with precisions and precision matrices rather than covariances and covariance matrices.

3 The joint likelihood is the product of the likelihood and prior and is proportional to the posterior distribution.

4 Throughout this paper, we use a parameterisation of the Wishart distribution over D×D matrices whereby $Wish\left(X|N,P\right)=K{\left|P\right|}^{N/2}{\left|X\right|}^{\left(N-D-1\right)/2}\;\exp\left[-\frac{1}{2}\text{tr}\left(XP\right)\right]$, where $K^{-1}=2^{ND/2}\pi^{D\left(D-1\right)/4}\prod_{d=1}^{D}\text{Γ}\left[\left(N+1-d\right)/2\right]$. Here *N* denotes the degrees of freedom and **P** is a symmetric, positive definite precision matrix.
