# Supplementary material for: A Bayesian spatial model for neuroimaging data based on biologically informed basis functions
Source: Neuroimage. 2017 Nov 1;161:134–48. doi: 10.1016/j.neuroimage.2017.08.009 (PMC5692833; doi:10.1016/j.neuroimage.2017.08.009)
Supplement: Supplementary file 1 [file mmc1.docx]

**Supplementary material for:**

**A Bayesian spatial model for neuroimaging data based on biologically informed basis functions**

Ismael Huertas,^1^ Marianne Oldehinkel,^2,3^ Erik S. B. van Oort, ^3^ David Garcia-Solis,^4,5^ Pablo Mir,^1,5^ Christian F. Beckmann,^2,3,6+^ Andre F. Marquand^2,3,7*+^

1: Instituto de Biomedicina de Sevilla (IBiS), Hospital Universitario Virgen del Rocío / CSIC / Universidad de Sevilla, Seville, Spain

2: Department of Cognitive Neuroscience, Radboud University Medical Centre, Nijmegen, the Netherlands

3: Donders Centre for Cognitive Neuroimaging, Donders Institute for Brain, Cognition and Behaviour, Radboud University, Nijmegen, the Netherlands

4: Servicio de Medicina Nuclear. UDIM. Hospital Universitario Virgen del Rocío, Seville, Spain

5: Centro de Investigación Biomédica en Red sobre Enfermedades Neurodegenerativas (CIBERNED), Spain.

6: Oxford Centre for Functional Magnetic Resonance Imaging of the Brain (FMRIB), University of Oxford, United Kingdom

7: Department of Neuroimaging, Centre for Neuroimaging Sciences, Institute of Psychiatry, King’s College London, United Kingdom

*: Corresponding Author. Andre Marquand, Donders Centre for Cognitive Neuroimaging, Donders Institute for Brain, Cognition and Behaviour, Kapittelweg 29, 6525 EN, Nijmegen, the Netherlands. Email: [a.f.marquand@fcdonders.ru.nl](mailto:a.f.marquand@fcdonders.ru.nl)

**+**: Shared senior author


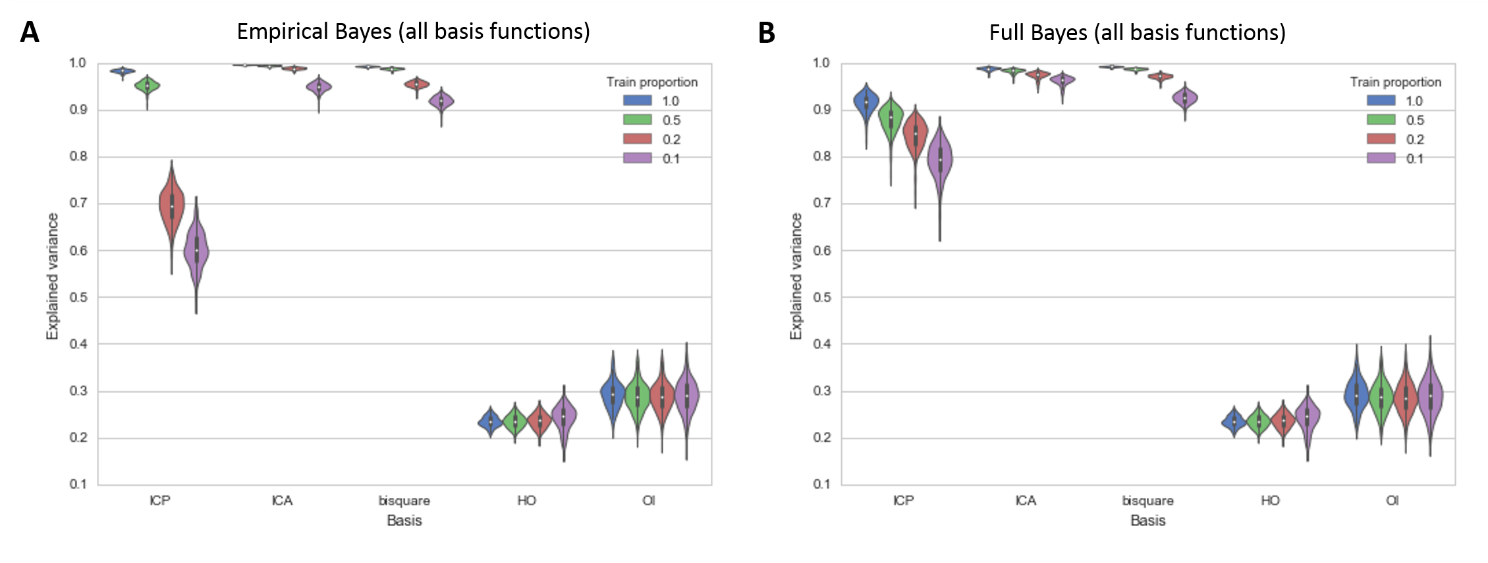


**Supplementary Figure 1.** Total variance explained by the Empirical Bayes approach (A) and the Full Bayesian approach (B) for models with a complete set of basis functions. ****

**Supplementary Figure 2:** Structure coefficients for each of the *M* basis functions (see text for details and compare with Figure 6). Each basis function was obtained by parcellating the striatum hierarchically into *d* = {2,…,30} parts. These different levels of the parcellation hierarchy are denoted by bars along the x-axis.
